# 3D-Printed Flow Cells for Aptamer-Based Impedimetric Detection of *E. coli* Crooks Strain

**DOI:** 10.3390/s20164421

**Published:** 2020-08-07

**Authors:** Ina G. Siller, John-Alexander Preuss, Katharina Urmann, Michael R. Hoffmann, Thomas Scheper, Janina Bahnemann

**Affiliations:** 1 Institute of Technical Chemistry, Leibniz University Hannover, Callinstraße 5, 30167 Hannover, Germany; siller@iftc.uni-hannover.de (I.G.S.); preuss@iftc.uni-hannover.de (J.-A.P.); scheper@iftc.uni-hannover.de (T.S); 2 Department of Environmental Science and Engineering, California Institute of Technology, 1200 E. California Blvd., Pasadena, CA 91125, USA; urmann.k@gmail.com (K.U.); mrh@caltech.edu (M.R.H.)

**Keywords:** additive manufacturing, impedimetric biosensor, aptasensor, screen-printed electrodes

## Abstract

Electrochemical spectroscopy enables rapid, sensitive, and label-free analyte detection without the need of extensive and laborious labeling procedures and sample preparation. In addition, with the emergence of commercially available screen-printed electrodes (SPEs), a valuable, disposable alternative to costly bulk electrodes for electrochemical (bio-)sensor applications was established in recent years. However, applications with bare SPEs are limited and many applications demand additional/supporting structures or flow cells. Here, high-resolution 3D printing technology presents an ideal tool for the rapid and flexible fabrication of tailor-made, experiment-specific systems. In this work, flow cells for SPE-based electrochemical (bio-)sensor applications were designed and 3D printed. The successful implementation was demonstrated in an aptamer-based impedimetric biosensor approach for the detection of *Escherichia coli* (*E. coli*) Crooks strain as a proof of concept. Moreover, further developments towards a 3D-printed microfluidic flow cell with an integrated micromixer also illustrate the great potential of high-resolution 3D printing technology to enable homogeneous mixing of reagents or sample solutions in (bio-)sensor applications.

## 1. Introduction

Electrochemical biosensors are widely used in areas such as health care, food control, or environmental analysis [1]. Especially the high selectivity of the biological recognition element and the sensitivity of electrochemical measuring methods (transducers) are potential key benefits of such sensors. Besides biorecognition elements like antibodies [2], lectin molecules [3], or bacteriophages [4], aptamers are of high interest [1]. In brief, aptamers are single-stranded nucleic acids that can display a high affinity to a target analyte. They are selected in vitro by an evolutionary process (SELEX, Systematic Evolution of Ligands by Exponential Enrichment) [5,6,7]. This class of biorecognition elements is often compared to antibodies. Aptamers benefit from their high stability in different chemical environments and to temperature changes [7]. Moreover, commercial, in vitro synthesis of nucleic acids enables a cost-efficient and flexible production and even allows site-specific modifications for attachment chemistries (e.g., thiol, amine, or biotin groups). Owing to their stability, consistent quality, small size, and broad possible target range, many assays can be designed utilizing aptamers as receptor molecules.

Electrochemical measurements, such as electrochemical impedance spectroscopy, offer label-free, rapid, and sensitive detection—a real benefit towards point-of-care testing. For instance, detection limits close to single-molecule detection have been reported (lipopolysaccharide concentration of 0.01 attomolar) [8]. Herein, we focused on impedimetric aptamer sensors. Previously reported targets include: Small molecules like progesterone [9] or Ochratoxin A (OTA) [10], proteins such as clinically relevant Vascular Endothelial Growth Factor (VEGF) [11,12] or carcinoembryonic antigen (CEA) [13], spore simulants of *Bacillus anthracis* [14], and various bacterial pathogens (e.g., *Salmonella typhimurium* [15,16], *Escherichia coli* (*E. coli*) [17,18,19,20], *Staphylococcus aureus* [21,22]). Infections with specific pathogenic *E. coli*, for instance, are the cause of multiple severe medical conditions [23]. Thus, rapid and reliable testing is needed to validate the safety of food or environmental samples. However, this presents only one example of why rapid detection methods are needed. Traditional methods based on bacterial culturing are laborious and take days, while specific amplification (e.g., polymerase chain reaction-based detection) needs only a few hours but does not differentiate between dead and living cells and requires costly devices and reagents. In comparison, electrochemical methods offer rapid and sensitive testing. Combined with aptamers as biorecognition element, the binding of whole cells enables selective detection of bacteria with no or little sample preprocessing. Besides electrochemical biosensors with antibodies and other biorecognition elements [24] with detection limits down to single-digit CFU (colony-forming units) mL^−1^, also a few electrochemical aptamer-based biosensors have been reported for the detection of *E. coli* [25,26,27]. However, they all have in common that electrodes are either self-made or custom-made, which hampers simple and open-access transfer to in-field applications, due to a lack of standardization of the electrode setup.

In recent years, an increasing number of publications—focused on screen-printed electrodes (SPE)—have been presented. SPEs are a disposable alternative to bulk electrodes. Besides in-house screen-printing facilities, different commercial suppliers are established, offering cost-efficient mass production [10,28,29]. Their products encompass disposable SPEs with different electrode materials (e.g., graphite, gold, platinum) or even nano-engineered surfaces (e.g., deposited gold nanoparticles or carbon nanotubes). Working electrodes with a diameter of only a few millimeters enable miniaturized assays [30].

Several authors state that a specific volume of reagents or sample of interest is placed onto the working electrode [31,32,33]. However, for many applications a static or dynamic flow cell might be of interest. Potential advantages include the handling of samples with defined volumes, a controlled environment, or, especially with regard to flow cells, an automation of experiments. A few authors mounted the electrode setup/screen-printed electrodes into dynamic flow cells or open/static cells. Polydimethylsiloxane (PDMS) [34,35] or 3D printing-based cells [36,37] are the most common, but also electrochemical cells based on glass [38], polypropylene [39], or poly(methyl methacrylate) (PMMA) [10] have been reported. Especially research laboratories may benefit from 3D printing as a flexible small-manufacturing tool not limited to flow cells or open cells. Instead, it opens access to a range of useful laboratory tools, while no special training is needed for the manufacturing process [40,41,42,43,44,45]. In brief, based on CAD (computer-aided design) modelling, a 3D model is structured into a finite number of layers to be printed layer by layer. In this way, a range of even complex 3D structures can be manufactured. The 3D-printed open cells may include a chamber for screen-printed microinterdigitated electrodes, which also enable a connection via Universal Serial Bus (USB) [30] and a chamber for bipolar electrodes [46]. An example of a 3D-printed microfluidic chamber was presented by Damiati et al. The SPE and the microfluidic chamber were bonded by a double-sided adhesive layer [37]. Moreover, there are some examples for open cells which are not based on 3D printing. Those include an open cell for the SPEs made of glass [38] and a one-compartment cell made of a not-further-specified material [47].

The potential of flow cells is illustrated by a study by Rhouati et al. [10]. They developed an automated aptamer-based detection of ochratoxin A in a custom PMMA flow cell for SPEs, which included a multiple-step processing of beer sample and reagents. For other flow cells, 3D printing has been utilized to manufacture master molds for PDMS and to provide (complex) housing for microfluidic channels made of PDMS [48].

In this study, we present 3D-printed cells that allow direct assembly of commercially available SPEs. Since there are different suppliers of SPEs of different size and shape, 3D printing offers customized integration of electrodes dependent on the specific needs for the experiments. The advantage of a static 3D-printed flow cell is demonstrated in an impedimetric and label-free aptamer-based biosensor approach for the detection of *E. coli* Cook’s strain as a proof of concept. An aptamer-based biosensing experiment involves several incubation and washing steps. Therefore, a system that allows the treatment of the SPE with defined volumes of liquids would be beneficial. In addition, for aptamer functionalization an exclusive exposure of only the working electrode is required. The static flow cell was designed and 3D-printed to meet these demands.

Moreover, this study also presents a 3D-printed microfluidic dynamic flow cell with an integrated micromixer, which can be operated automatically. Automated mixing of reagents or sample solutions before electrochemical measurements would not only ensure homogeneous mixing of the sample applied to the electrodes, but also contribute to reproducibility. A comparison of the mixing performance with and without micromixer unit in the flow cell demonstrates the great effect of integrated micromixers.

## 2. Materials and Methods

### 2.1. Design, 3D Printing, and Post-Processing

Flow cells were constructed with the computer-aided design (CAD) software SolidWorks (Dassault Systems, Waltham, MA, USA) and 3D printed using a high-resolution 3D printer (ProJet^®^ MJP 2500 Plus, 3D Systems, SC, USA). As printing material, a rigid translucent polyacrylate resin was used (VisiJet^®^ M2R-CL, 3D Systems, SC, USA) via MultiJet printing technology, involving a UV-curing process. The 3D parts were printed with a resolution of 800 × 900 dots per inch and a layer thickness of 32 µm. VisiJet^®^ M2-SUP functioned as the supporting material for the printing process. For removal of support material after printing, 3D-printed parts were placed for 15 min at −18 °C, then removed from the printing platform and placed for 45 min in a heat steam bath and paraffin oil bath at 65 °C (EasyClean unit, 3D Systems, SC, USA). Afterwards, the parts were rinsed in an ultrasonic water bath with the use of detergent (Fairy Ultra Plus, Procter and Gamble, CT, USA) for 30 min at 65 °C. The microfluidic structures of the dynamic flow cells were additionally flushed with paraffin oil (Carl Roth GmbH, Karlsruhe, Germany), soapy water, and water at the respective steps to remove remaining wax and oil residues.

Both constructed 3D-printed cell systems consisted of two parts—a bottom and a top part. Two exchangeable top parts were designed—one enclosing only the working electrode of the SPE and one enclosing all electrodes. For sufficient sealing, disc magnets (diameter: 6.4 mm, length: 1.6 mm; National Imports LLC, dba Magcraft, Vienna, VA, USA) and the corresponding electrodes enclosing O-rings (11 × 2 mm; 7 × 2 mm; Marco Rubber & Plastics, LLC, Seabrook, NH, USA) were embedded in the 3D-printed parts. The Standard Triangle Language (STL) files of the 3D-printed parts are provided as supporting information.

### 2.2. Biosensing Experiment in 3D-Printed Static Flow Cell

#### 2.2.1. Cultivation of Bacteria and Sample Preparation

*Escherichia coli* (*E. coli*) (Crooks strain, ATCC^®^ 8739, American Type Culture Collection, Manassas, VA, USA) was cultivated in appropriate nutrient broth and incubated at 37 °C, 150 rpm. To estimate the concentration of bacteria, optical density (OD) was measured at a wavelength of 600 (OD_600_) with a spectrophotometer. As the optical density (OD_600_) reached 0.5 (equals approximately 10^8^ cells/mL), 1-mL samples of the culture were taken and spun down in a regular lab centrifuge for 10 min at 5000× *g*. Supernatant was carefully discarded and the pellet was resuspended in 1 mL fresh nutrient medium. Centrifugation and washing steps were repeated twice and cells were diluted in ferri-/ferrocyanide buffer (FeSB) (see Section 2.2.4) to receive the required dilution.

#### 2.2.2. Preparation of Screen-Printed Electrodes (SPE)

Screen-printed gold electrodes (SPE) (DRP-220AT SPE) were purchased from Metrohm DropSens (Oviedo, Spain). The SPE consisted of a gold working electrode (4 mm in diameter), a gold counter electrode, and a silver pseudo-reference electrode and was made of high-temperature curing inks. The SPE was connected via a cable (CAC, Metrohm DropSens, Oviedo, Spain) to a potentiostat (VSP-300 with ultra-low current cable, BioLogic, Seyssinet-Pariset, France). All measurements were conducted at room temperature in an air-conditioned laboratory.

Before the start of a biosensing experiment, the SPE was mounted in the 3D-printed static cell with the top part enclosing all electrodes, cleaned, and activated according to the procedure described and optimized by Henihan et al. [49]. In brief, 100 µL of 0.1 mol L^−1^ sulfuric acid (H_2_SO_4_) was applied on all electrodes of the SPE. Then, 10 electrochemical cyclic voltammetry (CV) cycles with a voltage between 0 and 1.6 V, followed by three CV cycles with a voltage between 0 and 1.3 V, were run. After a biosensing experiment was done, the SPE was washed thoroughly with deionized nuclease-free water (Millipore Milli-Q^®^ system, Merck KGaA, Darmstadt, Germany) and cleaned with the same CV procedure.

#### 2.2.3. Aptamer Functionalization of the SPE

DNA oligonucleotides were purchased from Integrated DNA Technologies, Inc. (Coralville, IA, USA), with a thiol modification at 5. The oligonucleotide sequence of the used aptamer was described by Bruno et al. and was as follows: 5–ATC CGT CAC ACC TGC TCT GTC TGC GAG CGG GGC GCG GGC CCG GCG GGG GAT GCG TGG TGT TGG CTC CCG TAT–3 [50]. Until the start of an experiment, aptamer stock solution of 300 µM in deionized nuclease-free water was stored at 4 °C.

Chemical modification of oligonucleotides with thiol groups is a simple and often-used method for aptamer immobilization on gold surfaces [51]. Due to chemisorption of the activated thiol-modified aptamers on the gold surface, self-assembled monolayers were created.

For aptamer immobilization on the SPE, oligonucleotides were diluted to 1 µM in aptamer selection buffer (SB) containing 0.5 M sodium chloride (NaCl), 10 mM Tris buffer (Tris-hydrogen chloride (HCl)), 1 mM magnesium chloride (MgCl_2_); pH was set to pH 7.5. All buffer constituents used in this study were purchased from Sigma-Aldrich (St. Louis, MO, USA), unless noted differently. Furthermore, all buffer and solutions were diluted with deionized nuclease-free water. One µM aptamer solution was preconditioned with 200 µM Tris-(2-carboxyethyl)-phosphine-hydrochloride (TCEP) for 20 min for the reduction of disulfides, as described by Reich et al. [21]. Afterwards, the mixture was heated up to 95 °C for 5 min to break up hybridizations and potentially avoid incorrect folding. Cooling to room temperature then enabled the correct and functional folding of the oligonucleotides.

Only the working electrode should be functionalized with oligonucleotides. Thus, the top part of the 3D-printed cell was chosen, which was only enclosing the working electrode of the SPE.

When functionalizing the SPE with oligonucleotides, a balance has to be found between a high surface coverage density, which allows for a high capture capacity, and correct folding of the aptamer, which can be hampered at high immobilization density and, thus, hinder target binding [52]. Co-immobilization with mercaptohexanol (MCH) can be beneficial for a functional and well-ordered oligonucleotide monolayer. As a co-immobilized chemical, MCH has been found to support an upright position of immobilized DNA strands and can be used to control the aptamer density on the surface. Both contribute to correctly folded and functional aptamers. Another purpose is to fill in gaps on the gold surface. MCH blocks possible interaction sites and, thus, supports proper folding of the DNA oligonucleotides [21,53]. However, MCH can also displace aptamers. Therefore, a balance of concentration and incubation time has to be found [21]. The organization of the DNA strands to an ordered monolayer can take more than 8 h [21,54]. Therefore, to ensure a correct arrangement, the co-immobilization of aptamer and MCH was performed in an incubation step over night (for at least 15 h). Furthermore, in previous studies a ratio of 1:20 (1 µM aptamer and 20 µM MCH) was indicated as most suitable. Thus, this ratio was used for biosensing experiments [55].

In brief, reduced aptamer solution was mixed with 20 µM 6-mercapto-1-hexanol (MCH, 99%) for co-immobilization and 100 µL were applied on the working electrode in the corresponding 3D-printed static flow cell. As a precautionary measure, the system was placed in a wet chamber to additionally prevent evaporation. After immobilization was carried out at room temperature overnight, the solution was removed and electrodes were washed three times with SB. As an additional blocking step, the working electrode was exposed to 1 mM MCH for 15 min and subsequently washed again three times with SB [55].

**Figure 1:**
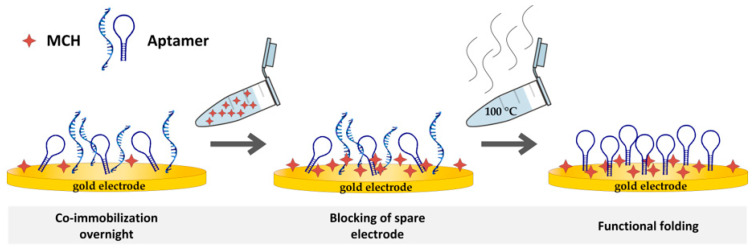
Schematic illustration of biosensor preparation. Co-immobilization step overnight, mercaptohexanol (MCH) blocking, and boiling step.

After the MCH blocking step, all electrodes were exposed to boiling water for 2 min to break up hybridizations and then washed three times with SB (see Figure 1). As illustrated in Figure 1, aptamer structures could still possess incorrect folding or interactions. Breaking up the hybridizations can rectify incorrect folding and, thus, nonfunctioning aptamers. The next step was the incubation in SB for 30 min at room temperature to promote correct folding of the aptamer. Subsequently, electrochemical impedance spectroscopy (EIS) measurements were performed (see Section 2.2.4).

#### 2.2.4. Biosensor Experiment with Bacteria in 3D-Printed Static Flow Cell

With the aptamer-functionalized SPE mounted in the 3D-printed static flow cell, kinetic EIS measurements were conducted. As described in previous studies, all EIS measurements were performed in ferri-/ferrocyanide buffer (FeSB) containing 2 mM potassiumhexacyanoferrate (II) and (III) (K_3_[Fe(CN)_6_]; K_4_[Fe(CN)_6_], equimolar) in SB [21,49]. For all biosensor measurements, the top part enclosing all electrodes for the 3D-printed static flow cell was used. Impedance was measured at open circuit potential at an amplitude of 10 mV Root Mean Square (RMS) at 7 logarithmic spaced frequencies per decade in the range of 200.000–0.1 Hz, as these parameters have proven to be suitable in previous studies related to this subject [21,49]. Measurements were repeated four times, whereas the last three cycles were included in fitting and analysis. Spectra were fitted to the modified Randles circuit, which describes the present circuit between the electrodes [3]. Here, R_sol_ represents the solution resistance, R_ct_ is the charge transfer (interface) resistance, W represents the Warburg element (diffusion of ions to electrodes), and CPE is the constant phase element for the double layer at the surface. R_ct_ was chosen for further analysis since a binding event on the surface especially affects the charge transfer [21,49,56].

As a control for the biosensing experiment, electrochemical impedance was measured of the aptamer-functionalized SPE (before *E. coli* samples were applied on electrodes). Afterwards, electrodes on the SPE were washed with SB and dried before the prepared *E. coli* cell sample (100 µL) was applied to the working electrode and incubated for 1 h. Electrodes were thoroughly washed three times with SB and EIS was performed subsequently.

For regeneration of the aptamer-functionalized SPEs, the bacterial sample was removed, electrodes were washed thoroughly with SB, and the surfaces were exposed to boiling water for 2 min. After washing steps and incubation in SB (30 min, room temperature), SPEs were ready for another biosensing experiment.

### 2.3. Experiments in Dynamic 3D-Printed Microfluidic Flow Cell

The 3D-printed flow cell presented here features a micromixer upstream of the system, which ensures a homogeneously mixed solution before it reaches the electrodes. The mixer, known as HC-mixer, makes use of a split-and-recombine technique and H-shaped channel modules to intensify and enhance mixing performance [57,58]. The mixer was designed according to Enders et al. and connections to the tube and pump system were set up [58]. Right behind the micromixer unit, a fluid chamber enclosing the relevant electrodes of the SPE was integrated. The fluid chamber enclosing all electrodes of the SPE holds a volume of 30 µL. For connection of the microfluidic system to syringes and the pump system, the design of the system was adapted to the dimensions of a Dolomite 4-way microfluidic connector (Dolomite Center Ltd., Royston, UK). A syringe pump (AL-100, World Precision Instruments, Sarasota, FL, USA) was taken for this study.

The mixing performance of the integrated micromixer unit was determined by computational fluid dynamics (CFD) simulation experiments and compared with a reference design (T-mixer). CFD simulations were performed using COMSOL Multiphysics 5.4 (COMSOL Inc., Stockholm, Sweden). For the simulation, an aqueous liquid with a corresponding density (1 g cm ^−1^) and dynamic viscosity (1000 × 10^6^ kg m^−1^ s^−1^) was set. Flow rate of 3 µL s^−1^ for both inlets was chosen and a laminar flow as present in microfluidic channels was assumed. To simulate mixing, a solute concentration of 1 mol m^−3^ at one inlet and 0 mol m^−3^ at the other inlet was defined. A diffusion coefficient of 10^−9^ m^2^ s^−1^ was set, presenting a commonly used constant [58,59]. Remaining simulation settings were chosen according to Enders et al. [58].

Practical experiments with the two-mixer designs (T-mixer and HC-mixer) were performed to confirm the results of the simulations. Acidic 100 mM sodium dihydrogen phosphate and basic 50 mM di-potassium hydrogen phosphate (both from Carl Roth GmbH, Karlsruhe, Germany) with 50% (v/v) (volume concentration) bromothymol blue solution (Sigma-Aldrich, St. Louis, MO, USA) were used to visualize mixing. Bromothymol blue indicates acidic pH by a yellow color, neutral pH by a green color, and basic pH by a blue color. In selecting the concentrations, care was taken to ensure that equal proportions of both solutions resulted in a neutral pH value (green). For the experiments, a flow rate of 3 µL s^−1^ was set at each inlet for 1 min to ensure a steady state. Pictures were taken using a digital microscope (VHX-6000 digital microscope, KEYENCE Deutschland GmbH, Neu-Isenburg, Germany). The magnification was set to 30×.

## 3. Results and Discussion

### 3.1. Introduction of Static 3D-Printed Flow Cell

**Figure 2:**
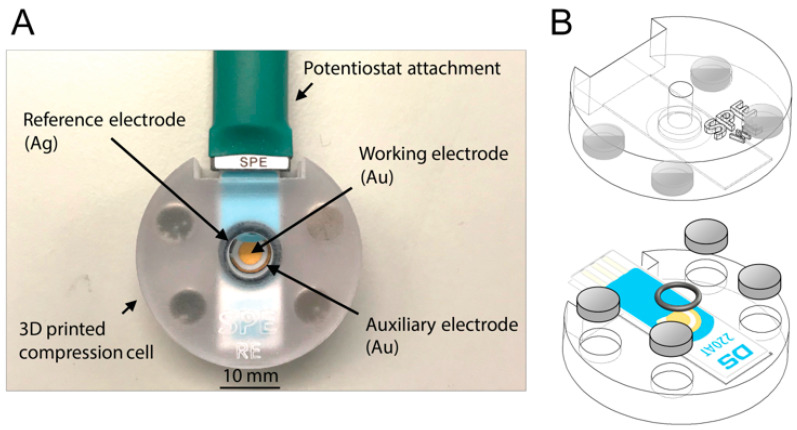
Image of the 3D-printed static flow cell with mounted screen-printed electrode (SPE) and connected cable (**A**) and illustration of the setup (here with top part enclosing only the working electrode of SPE) (**B**).

For performing biosensing experiments with SPEs, a static cell was designed and 3D printed. It consisted of two parts—a bottom and a top part. Both parts held a slot specifically adapted to the dimensions of the SPE that was providing the necessary support and ensured a firm fit in the static flow cell. The top part included a cavity, focusing the corresponding electrodes of the SPE, which enabled the exposure of electrodes to a defined volume of liquid, such as buffer solutions or bacteria samples. During the biosensing experiment, the top part of the static flow cell has to be exchanged. Only the working electrode should be functionalized with aptamers, whereas for electrochemical measurements all electrodes have to be in contact with the respective buffer solution. Therefore, two exchangeable top parts were designed—one enclosing only the working electrode of the SPE and one enclosing all electrodes. Changing the top parts required easy (dis)assembly while holding sufficient sealing at the same time. This was guaranteed by magnets and an O-ring, which was perfectly enclosing the corresponding electrodes (see Figure 2).

3D printing technology not only opened the door to the production of small batches or prototypes in a short amount of time, it also allows researchers to create individually designed experimental setups and devices of almost unlimited complexity, leaving them no longer reliant on commercially available goods. There are several reports on custom cell systems, for example, made of methyl methacrylate material or glass, that supported electrochemical measurements [10,60]. However, traditional manufacturing methods are often disadvantageous as they involve time-consuming process steps that often require special training, resulting in an overall laborious and cost-intensive process.

The 3D printing material used in this study is considered to be (in vitro) biocompatible, as has been shown in previous publication [61]. Therefore, the material is suitable for biotechnological applications. Furthermore, the chemical stability against ethanol or isopropyl alcohol solvents, which are frequently used in the laboratory for material disinfection, was also shown [61]. Since a variety of different 3D printing materials are already commercially available, compatible material with suitable properties can be selected for the most diverse applications and experimental requirements. Autoclavable 3D printing materials are also available, ensuring sterility of 3D-printed parts for biological applications.

### 3.2. Biosensing Experiments

**Figure 3:**
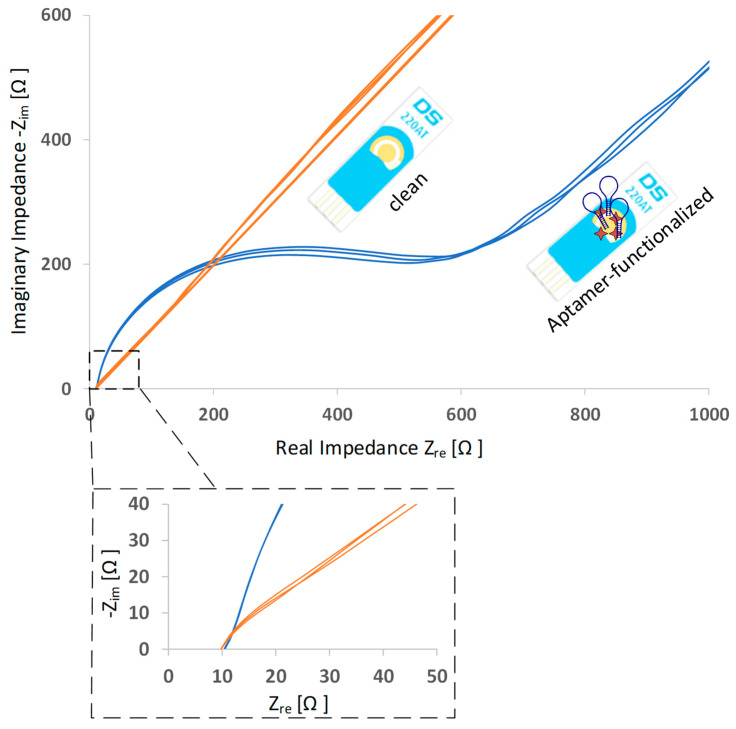
Typical electrochemical impedance spectra (Nyquist plots) before and after aptamer immobilization. The number of spectra curves in one graph indicate the number of EIS cycles.

To verify the successful aptamer immobilization on the working electrode, EIS measurements were conducted. Impedance is a mathematically complex value that consists of a real and an imaginary part. A common presentation of the data is to plot the real and imaginary parts against each other (Nyquist plot). Each point on the plot presents the impedance parts at one frequency. A comparison of Nyquist plots before and after aptamer functionalization is shown in Figure 3. A significant increase in impedance can be observed after aptamer is immobilized. Due to the immobilized oligonucleotides on the gold surface, the electron transfer is hindered and impedance increases.

**Figure 4:**
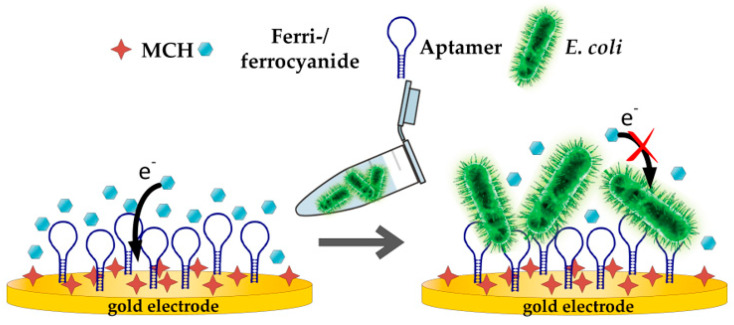
Schematic illustration of aptasensor experiment.

The general principle of an aptamer-based impedimetric detection of a target involves a redox probe, which is supplied in a buffer solution. The redox probe is responsible for the electron transfer from the working electrode to the counter electrode. Here, the ferri-/ferrocyanide couple was used as a redox probe due to its fast electron transfer rate [62]. To assess the applicability of the 3D-printed static flow cell in combination with the SPE for detection of *E. coli* Crooks strain, direct capture experiments with *E. coli* were performed as proof of concept (see Figure 4).

**Figure 5:**
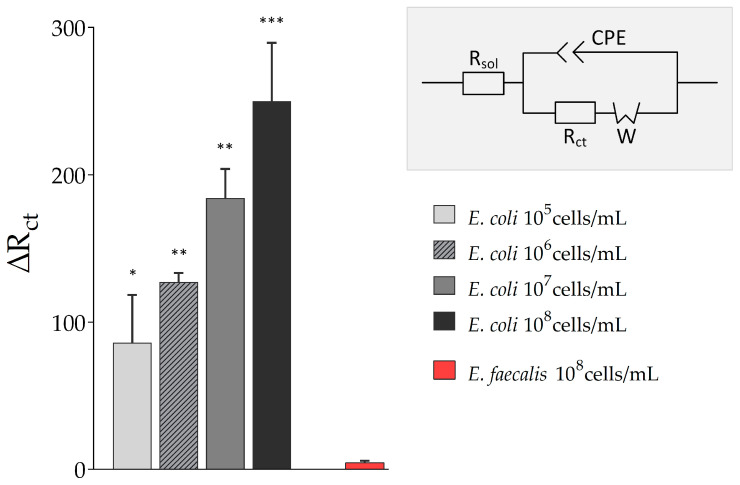
Results of biosensing experiments. Electrochemical Impedance Spectroscopy (EIS) measurements of biosensor experiments with varying *Escherichia coli* Crooks and *Enterococcus faecalis* (*E. faecalis*) concentrations were fitted to the modified Randles circuit with R_sol_: solution resistance, R_ct_: charge-transfer resistance, W: Warburg impedance, and CPE: constant phase element. Extracted charge transfer resistance ∆R_ct_ is displayed. All experiments were repeated several times in independent experiments (technical replicates, n = 3) and significant differences to *Enterococcus faecalis* as a control are indicated: * *p* < 0.05, ** *p* < 0.01, *** *p* < 0.001.

After the SPE was functionalized and prepared, samples with varying *E. coli* Crooks strain concentrations were loaded onto the electrodes by means of the 3D-printed static flow cell and EIS was conducted. As mentioned in Section 2.2.4. electrochemical impedance data were fitted to the modified Randles circuit, subsequently, and the charge transfer (interface) resistance (R_ct_) was chosen as parameter for analysis (see Figure 5). It was expected that upon binding of *E. coli* Crooks strain, the electron transfer between electrode and buffer solution containing the redox probe was influenced and that this was reflected in an altered R_ct_.

The charge transfer resistance (R_ct_) increased with the concentration of *E. coli* in the sample solution (from ∆R_ct_ = 86.25 ± 32.31 at 10^5^ cells/mL *E. coli* to ∆R_ct_ = 249.99 ± 39.58 at 10^8^ cells/mL *E. coli*). The more *E. coli* cells that were present in the sample, the higher and more substantial was the influence on the electron transfer between electrode and redox probe. However, even low concentrations of *E. coli* Crooks strain were detectable. In addition, no cross-reactivity of the aptamer used in this study was observed, here to *Enterococcus faecalis*, and other previous published work already reported no or no significant binding of the used aptamer to *Staphylococcus aureus*, *Salmonella typhimurium*, or *E. coli* K12 [25].

**Figure 6:**
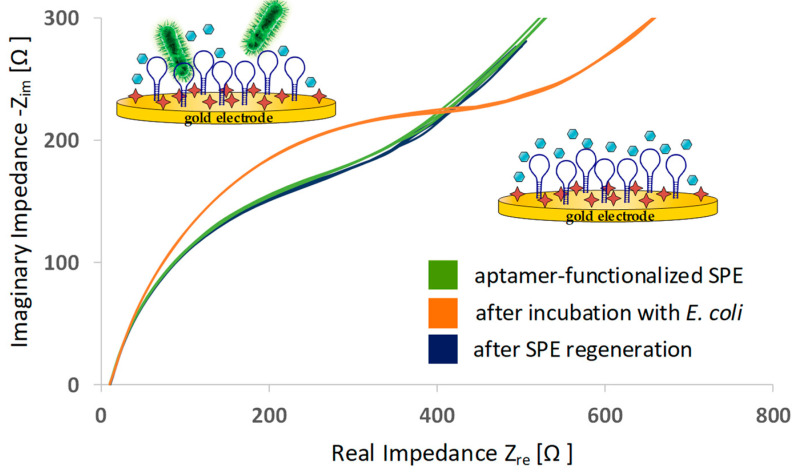
Typical electrochemical impedance data (Nyquist plots) of aptamer-functionalized SPE, after a biosensing run with *E. coli* and after regeneration of the SPE. The number of spectra curves in one graph indicates the number of EIS cycles.

After one biosensing run was done, the aptamer-functionalized SPE was regenerated in a cleaning step with boiling water. Thereby, aptamer structures unfold and potential captured targets (here, whole *E. coli*) can be released. A subsequent incubation step in aptamer selection buffer allowed the oligonucleotides to fold again in functional conformation and the biosensor was ready for another biosensing experiment. Nyquist plots of EIS indicated that the regeneration step successfully restored the SPE: The electrochemical condition of the regenerated SPE was identical to the status before sample incubation (see Figure 6). Aptamer structures were successfully regenerated.

The custom-designed, 3D-printed static flow cell has proven to be a prerequisite for performing the biosensing experiment—the 3D-printed system not only provides a stable fit of the SPE and thereby facilitates the handling, it also enables accurate washing and incubations with defined volumes of liquids. An exchangeable top part allows for the selective treatment of only the working electrode or all electrodes by the choice of top. Without this feature, a biosensing experiment following the approach of this study would have been difficult to carry out.

In all, aptamer functionalization of the mounted SPE and subsequent direct capture experiments with *E. coli* Crooks strain were successfully performed in a 3D-printed static flow cell. Due to the electrochemical detection method, bacteria were detected label-free, without the need of laborious and costly labeling procedures. With the use of aptamers as biological recognition element, a highly selective and sensitive biosensor was created. The aptamer used in this study has already been intensively characterized [25,26,63] but, to the best knowledge of the authors, it has never been used in combination with a 3D-printed chamber. The 3D printing material, in general and particularly the customized 3D-printed static flow cell, have proven to be expedient and beneficial in the context of electrochemical sensors. In fact, the 3D-printed static cell could find application in all kinds of SPE-based electrochemical studies.

### 3.3. Investigation of 3D-Printed Microfluidic Dynamic Flow Cell for SPE Applications

The 3D-printed static flow cell introduced in this study is based on manually performed washing and incubation steps. However, an automated flow cell system may be profitable in other aspects or for other applications. There are several reports demonstrating the advantages of flow systems for SPE applications [10,64]. A consistent, continuous liquid flow may improve washing or lower the limit of detection (LOD) of aptasensors. In addition, an automated system has great potential to improve reproducibility and lower the workload. Here, 3D printing technology offers tremendous potential for the rapid fabrication of complex flow systems that can be integrated in pump systems for automation.

**Figure 7:**
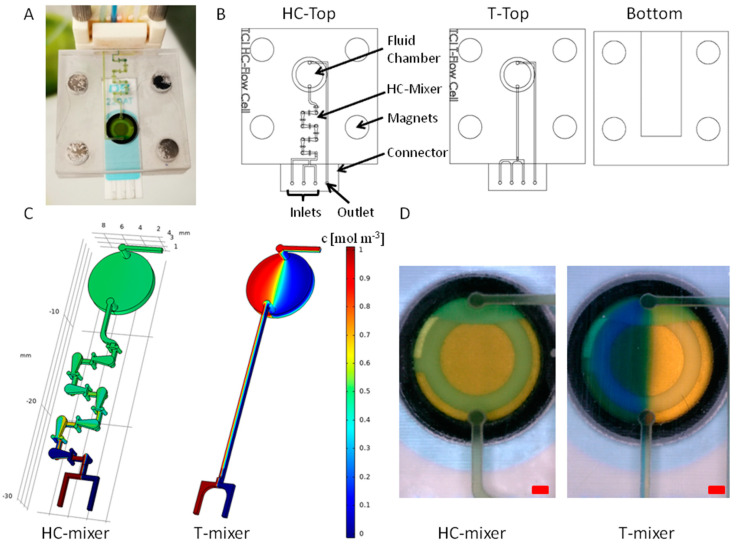
(**A**) Image of a 3D-printed dynamic flow cell with an integrated SPE. (**B**) computer-aided design (CAD) drawings of the top parts with HC-mixer and T-mixer and the bottom part of the flow cells. (**C**) COMSOL simulation of the interior structure of the dynamic flow cells (inlet flow rates of each 3 µL s^−1^). The left inlet and the right inlet were simulated with concentrations of 1 mol m^−3^ and 0 mol m^−3^, respectively (see legend). (**D**) Pictures of the fluid chambers during mixing experiments with flow rates of 3 µL s^−1^ for both inlets. Yellow-colored solution was pumped into the left inlet and blue-colored solution into the right inlet. The red scale bar represents 1 mm width.

A 3D-printed dynamic flow cell for SPE applications is shown in Figure 7A. The flow cell has three inlets and an outlet, which can be connected directly to sample and reagent solutions via tubing. The inflow and outflow into the flow cell can thus be controlled and enables reproducible and continuous sample processing. To enable mixing-dependent applications with the flow cell, a microfluidic HC-mixer was integrated upstream of the fluid chamber and the mixing performance was compared with an integrated T-mixer, referring to a T-shaped channel module [58].

The mixing performance of the integrated micromixer units was analyzed in both by CFD simulation and in practical experiments. For an overview, Figure 7A,B shows a picture of an assembled dynamic flow cell and CAD drawings of the individual parts, respectively. The mixing efficiency was determined by simulation (see Figure 7C). A homogeneous mixing is shown as green (0.5 mol m^−3^), while no mixing is shown as blue (0 mol m^−3^) and red (1 mol m^−3^). After four of seven subunits of the HC-mixer, a homogeneous mixing was evident. The simulation with a T-mixer showed that there was almost no mixing of the liquids within the channel. Therefore, most parts of the liquid chamber appeared blue and red. This is due to the laminar flow present in microfluidic channels, which is why diffusion is the only mixing effect in straight microfluidic channels [58]. These results were confirmed by practical experiments. Since a homogeneous mixing with equal proportions of the solutions used resulted in a neutral pH value, a color change from blue or yellow to green is an indicator of successful mixing. The integration of an HC-mixer resulted in a uniform green color inside of the fluid chamber (see Figure 7A,D). In contrast, the T-mixer showed a mostly two-part segmentation of the liquids (right side is yellow, left side is blue). Between the segments, a narrow, green transition area was visible, which indicates a certain diffusive mixing, but not a homogeneous mixing within the fluid chamber of the flow cell.

Both the simulation and the practical experiments demonstrated the excellent and superior performance of the HC-mixer. The HC-mixer ensures a rapid and homogeneous mixing and is, therefore, of great interest for integration into 3D-printed flow cells–especially when using small sample volumes. A possible application of the flow cell presented here is the automated and time-controlled incubation of buffers, aptamer, and sample solutions. Concentration gradients can be run or sample concentrations can be adjusted automatically. The integrated micromixer allows for a fast and efficient mixing of reagents and/or mixing of the sample before it comes into contact with the electrodes of a mounted SPE. In future work, sensor experiments with real samples have to be performed to validate the functionality of the 3D-printed dynamic flow cell.

## 4. Conclusions

This work demonstrates the successful development of a 3D-printed static flow cell for SPE-based applications. An aptamer-based impedimetric detection of *E. coli* Crooks strain as a proof of concept served to demonstrate the successful implementation of the system. With the use of commercially available SPEs, we presented not only a cost-effective and disposable alternative to bulk electrodes, but, moreover, demonstrated regeneration and reuse of the biosensor. Electrochemical biosensing experiments performed in 3D-printed static flow cell showed sensitive and selective binding to the target, even after multiple SPE regenerations. In summary, the direct capture biosensor presented rapid and sensitive detection without the need of costly labeling procedures or laborious sample preparation. The customized 3D-printed static flow cell proved to be indispensable for performing a biosensing experiment. The intensive/various washing and incubation steps required would have been difficult to perform without the 3D-printed system. In fact, the 3D-printed cells could serve as a fundamental base for SPE-based biosensors of any kind. The basic design allows easy fabrication with almost all 3D printing technologies and also allows researchers to customize the system to their applications and specifications.

A dynamic, 3D-printed microfluidic flow cell was presented, which enabled automated control of the fluid flow. By integration of a micromixer unit into the design, a homogeneous mixing of solutions prior to electrochemical measurement was provided. This not only replaces intensive, manually performed mixing steps, but also contributes to reproducible measurements and experiments. In combination with an automated pumping system, the fluid flow can be controlled from continuous (e.g., for washing the electrodes) to paused flow (e.g., to allow time-dependent incubations).

Compared to commercially available static and dynamic flow cells, the 3D-printed cells presented here are much more cost-effective. Depending on the 3D printing technology and printer system used, fabrication and labor costs may vary. The production of the ready-to-use 3D-printed cells in the proposed printer system cost a total of about $18, taking into account material consumption and all consumables used, whereas comparable commercially available systems made of polytetrafluoroethylene (PTFE) are more than 50 times more expensive. As the operation of most 3D printers and the design of objects with CAD software is easy to use and does not require special training, the entire manufacturing process can be carried out with little training. In addition, 3D printing technology enables fabrication of experiment-specific devices on demand, directly on site in the laboratory. In case no 3D printer is available on site, commercial or academic 3D printing services offer contract printing at low cost.

This study demonstrated the great potential of customizable 3D-printed devices. Holding almost unlimited design options, 3D printing technology is of incredible interest for rapid fabrication of experiment-specific labware, test equipment, or whole test systems. With immense flexibility, 3D printing technology provides a platform for individual adaptations or system integrations, as well as the parallelization of complex systems. That way, not only electrochemical biosensing can benefit from 3D printing technology, but all scientific disciplines.
